# Supplementary material for: Single-Cell Sequencing Reveals an Intrinsic Heterogeneity of the Preovulatory Follicular Microenvironment
Source: Biomolecules. 2022 Jan 29;12(2):231. doi: 10.3390/biom12020231 (PMC8961562; doi:10.3390/biom12020231)
Supplement: Supplementary file 1 [file biomolecules-12-00231-s001.zip › biomolecules-1524839-supplementary.pdf]

Table S1 The characteristic and clinical outcomes of patients

	**Patient 1**	**Patient 2**	**Patient 3**	**Patient 4**	**Patient 5**	**Patient 6**
Age	27	34	31	23	33	30
protocol	GnRH-a	GnRH-ant	GnRH-ant	GnRH-ant	GnRH-ant	GnRH-a
Total Gn dose (IU)	1500	1000	2075	1802.5	2150	1525
Days of Gn stimulation	10	8	13	15	9	12
Trigger	Ovidrel	Decapeptyl+ HCG	Decapeptyl+ HCG	Decapeptyl+ HCG	Ovidrel	Ovidrel
P on HCG day (ng/ml)	0.75	1.16	0.88	0.62	0.41	0.7
LH on HCG day (mIU/ml)	0.24	1.1	0.95	1.57	2.37	0.59
E2 on HCG day (pg/ml)	1568	2416	2800	1159	2626	4334
P after HCG day (ng/ml)	10.48	11.28	11.56	2.05	2.83	-
LH after HCG day (mIU/ml)	-	20.62	38.26	22.45	-	-
HCG after HCG day (IU/L)	97.25	90.62	68.26	47.32	57.02	141.88
E2 after HCG day (pg/ml)	1885	2941	2629	1849	4734	3939
oocyte	MII	MII	MII	MII	MII	MII
Embryo score[1]	1 cell stage	3BB	3BB	4BB	332	4BB

Abbreviations: Gn, gonadotropin; P, progesterone; HCG, human chorionic gonadotropin; LH, luteinizing hormone; E2, estrodial; GnRH-a: gonadotropin releasing hormone agonist; GnRH-ant: gonadotropin releasing hormone antagonist; MII: meiosis II stage.

[1] High quality embryo was defined as embryos with 7-9 cells and <30% fragmentation on day 3 or blastocysts meet or exceed 3BB by Gardner score

Blanks indicate that the patient did not take the indicated tests.

Table S2 The sequencing results of each sample before and after quality control

	Patient 1	Patient 2	Patient 3	Patient 4	Patient 5	Patient 6
Before quality control						
Cell number	1786	739	3246	3544	3671	2879
Cell viability(%)	85.00	95.00	95.00	68.00	83.50	79.24
Mean reads per cell	127265	112733	129462	155119	116171	111277
median UMI	3982	3941	2434.5	7289	9026	11975
median gene	1602.5	1463	1165	2390	2905	3327
percent.mt >20%(%)	2.184	12.855	0.062	1.044	0.899	1.806
percent.mt >50%(%)	0.504	2.571	0	0.056	0	0.139
After quality control						
Cell number	1712	705	3013	3414	3307	2441
median UMI	3928	4028	2408	7097	8298	10431
median gene	1588.5	1519	1150	2345	2770	3089
percent.mt >20%(%)	1.752	10.638	0.067	1.025	0.998	1.925
percent.mt >50%(%)	0	0	0	0	0	0

Table S3 Numbers of different cell type for each sample

	Patient 1	Patient 2	Patient 3	Patient 4	Patient 5	Patient 6
GCs	334	218	281	3286	3263	2232
Macrophages	937	200	2301	35	2	126
DCs	180	171	329	7	6	61
T cells	144	34	58	5	9	10
Neutrophils	112	5	44	4	1	2
Epithelial cells	5	77	0	77	26	10

Abbreviations: GCs, granulosa cells; DCs, dendritic cells.

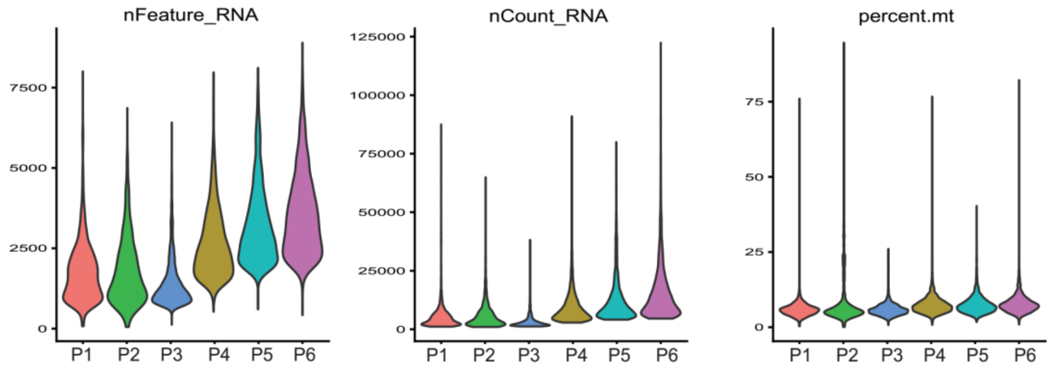

Supplementary Figure S1 Violin plots showing the number of genes, the number of UMI and the percentage of mitochondrial gene filter by samples.

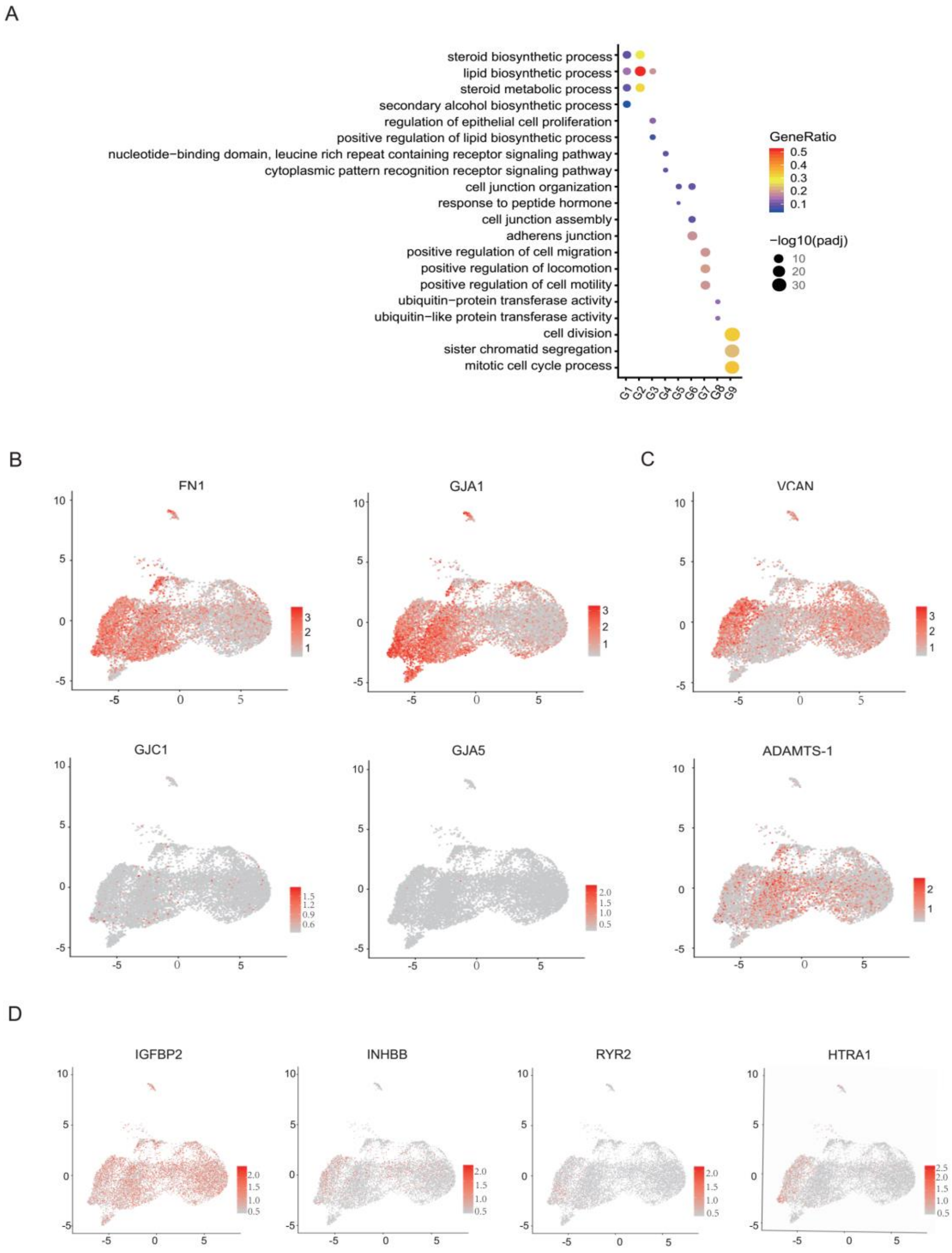

Supplementary Figure S2 (A) Representative GO terms enriched in the nine clusters in GCs (B) UMAP plot showing the expression patterns of GJA1, FN1, GJC1 and GJA5 in GCs. (C) UMAP plot showing the exclusive expression patterns of VCAN and ADAMTS1 in GCs. Cells in (B) and (C) are colored with the expression levels of the indicated genes, with a gradient

from gray to red indicating the lowest to the highest gene expression level. (D) UMAP plot showing the expression of marker genes of CCs and MGCs.

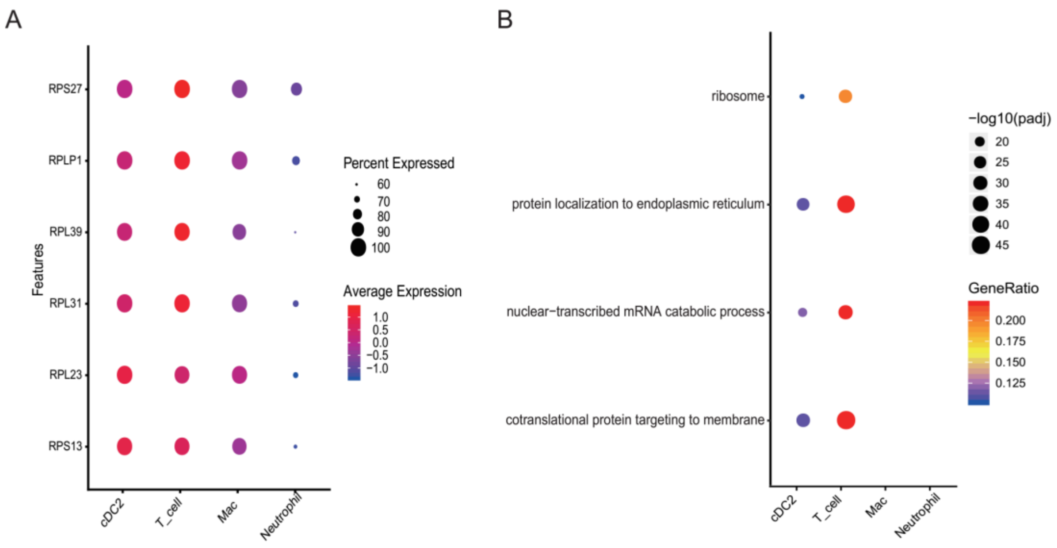

Supplementary Figure S3(A) Violin plots showing the expression levels of highly expressed genes in DCs and T cells. (B) Representative GO terms enriched in DCs and T cells.
